# Improve the performance of CT-based pneumonia classification via source data reweighting

**DOI:** 10.1038/s41598-023-35938-3

**Published:** 2023-06-09

**Authors:** Pengtao Xie, Xingchen Zhao, Xuehai He

**Affiliations:** 1 Department of Electrical and Computer Engineering, University of California San Diego, San Diego, USA; 2 Department of Electrical and Computer Engineering, Northeastern University, Boston, USA; 3 Department of Computer Science and Engineering, University of California Santa Cruz, Santa Cruz, USA

**Keywords:** Viral infection, Computed tomography

## Abstract

Pneumonia is a life-threatening disease. Computer tomography (CT) imaging is broadly used for diagnosing pneumonia. To assist radiologists in accurately and efficiently detecting pneumonia from CT scans, many deep learning methods have been developed. These methods require large amounts of annotated CT scans, which are difficult to obtain due to privacy concerns and high annotation costs. To address this problem, we develop a three-level optimization based method which leverages CT data from a source domain to mitigate the lack of labeled CT scans in a target domain. Our method automatically identifies and downweights low-quality source CT data examples which are noisy or have large domain discrepancy with target data, by minimizing the validation loss of a target model trained on reweighted source data. On a target dataset with 2218 CT scans and a source dataset with 349 CT images, our method achieves an F1 score of 91.8% in detecting pneumonia and an F1 score of 92.4% in detecting other types of pneumonia, which are significantly better than those achieved by state-of-the-art baseline methods.

## Introduction

Pneumonia is a life-threatening disease caused by bacteria, virus, and fungi. It is a type of acute respiratory infection in the lungs. According to the World Health Organization, pneumonia caused about 0.74 million deaths of children in 2019, which accounts for 14% of all deaths of children under 5 years old^1^. In particular, the recent COVID-19 pneumonia caused 6.5 millions deaths globally. Chest computed tomography (CT) scans are broadly used to diagnose pneumonia (including COVID-19 and other types of pneumonia)^2–4^, differentiate different types of pneumonia^5^, assess the severity of pneumonia^6^, etc. In medically under-served areas such as rural areas, well-trained radiologists who can accurately interpret CT scans to detect and assess the severity of pneumonia are lacking. To assist radiologists in accurately and efficiently detecting pneumonia and distinguishing different types of pneumonia from CT scans, many deep learning methods have been developed^7–22^. For example, Qian et al.^7^ proposed a multi-task multi-slice deep neural network to screen pneumonia from CT scans. Abdel-Basset^12^ developed a two-stage deep learning method to distinguish community-acquired pneumonia from COVID-19 pneumonia based on CT scans. Ortiz^23^ developed a deep neural network which leverages CT scans and clinical metadata to distinguish COVID-19 pneumonia from other viral pneumonia.

Deep neural networks typically have a large number of weight parameters. To effectively train them, large amounts of CT scans with pneumonia annotations are needed. Due to data privacy concerns and high costs of annotating pneumonia, it is very difficult to obtain a large number of annotated CT scans. Without sufficient training data, deep neural networks perform unsatisfactorily on test cases. One way of addressing this problem is transfer learning (TL)^24^, which leverages data from source domains to help train a target model in a target domain. It is often the case that some source examples have low quality and should be down-weighted. For example, some source examples are noisy and some examples have large domain discrepancy with target data. It is important to automatically identify such low-quality source examples and down-weight them during the transfer learning process. Many methods^25–33^ have been developed for source example reweighting. These methods do not learn weights of source examples by maximizing the performance of the target model on a held-out validation set. As a result, the target model trained (implicitly) using reweighted source examples is not guaranteed to have good generalization performance on test data. Bi-level optimization (BLO)^34^ based approaches^35–40^ have been proposed for data reweighting by explicitly minimizing a validation loss, where the first-level trains network weights on a training dataset and the second level learns data weights on a validation set. Applying these approaches for source data reweighting in TL, it is required to train source model and target model in a multi-task learning (MTL) way at the first level. As noted by Zhuang et al.^24^, MTL formulations are not suitable for TL since MTL focuses on training both source and target models well while the goal of TL is to train the target model well.

To address the problems of existing methods, we propose a three-level optimization based method which performs three learning stages end-to-end. At the first stage, a source model is trained by minimizing weighted losses on source examples. Source example weights are tentatively fixed at this stage. At the second stage, we train a target model by transferring knowledge from source to target. We propose a new ranking-based knowledge transfer approach that allows source model and target model to have different architectures, different dimensions of encodings, different weight parameters, etc. Our method uses the source model to generate a ranking on target examples. Then we train the target model by letting it predict the generated ranking. At the third stage, we evaluate the trained target model on a validation set. Note the validation loss is a function of source example weights. We update weights of source examples by minimizing the validation loss.

Compared with existing methods, our method has the following advantages: (1) our method learns source example weights by explicitly minimizing the validation loss of the target model, which therefore can make the target model generalize well on test data; (2) our method focuses on improving the target model by transferring knowledge from source to target, instead of training source and target models in a multi-task learning framework.

We apply the proposed method to detect pneumonia and distinguish COVID-19 and other types of pneumonia, on a target dataset with 2218 CT scans and a source dataset with 349 CT images. Our method achieves an F1 score of 91.8% for COVID-19 pneumonia and an F1 score of 92.4% for other types of pneumonia. Our method outperforms state-of-the-art baseline methods.

The major contributions of this paper are as follows:

- To accurately detect pneumonia from CT scans and distinguish COVID-19 pneumonia and other types of pneumonia, we propose a three-level optimization based method which leverages external CT data from a source domain to help train a target model and automatically down-weights low-quality source data examples. Our framework learns source example weights by explicitly minimizing the validation loss of the target model and performs knowledge transfer from source to target instead of training source and target models simultaneously in a multi-task learning way.
- We propose a new ranking-based knowledge transfer approach where a source model generates a ranking and a target model predicts this ranking.
- We demonstrate the effectiveness of the proposed method in detecting pneumonia from CT scans and differentiating COVID-19 pneumonia and other types of pneumonia. By leveraging a source dataset with 349 CT images, our method achieves an F1 score of 91.8% for COVID-19 and an F1 score of 92.4% for other types of pneumonia on a target dataset with 2218 CT scans. Our method outperforms state-of-the-art baseline methods significantly.

## Related works

### Deep learning for detecting pneumonia from CT scans

Many deep learning methods have been developed for detecting pneumonia from CT scans. Qian et al.^7^ proposed a multi-task multi-slice deep neural network to screen pneumonia from CT scans. Li et al.^8^ developed a deep learning method to analyze thick-section CT scans for assessing the severity and progression of COVID-19. Amyar et al.^9^ proposed a multi-task deep learning method to identify and segment COVID-19 from CT scans. Ni et al.^10^ developed a deep neural network for COVID-19 pneumonia classification, segmentation, and localization from CT scans. Zhang et al.^41^ leveraged a human-in-the-loop training strategy to learn a segmentation network for quantifying the volumes of COVID-19 infection on CT scans. Ko et al.^42^ proposed a transfer learning method which leverages pretrained convolutional neural networks to detect COVID-19 pneumonia from a single chest CT image. Maghdid et al.^43^ used pretrained deep convolution network to detect pneumonia from CT scans and chest X-rays. Xu et al.^11^ developed a 3D deep convolutional network model to distinguish COVID-19 from influenza-A viral pneumonia and healthy cases from CT scans. Abdel-Basset^12^ developed a two-stage deep learning method to distinguish community-acquired pneumonia from COVID-19 pneumonia based on CT scans. Chen^44^ applied UNet++ to detect COVID pneumonia from CT scans. Zhou et al.^45^ applied YOLOv3 to differentiate novel coronavirus pneumonia from influenza pneumonia based on CT scans. Chaudhary et al.^46^ developed a two-stage convolutional neural network (CNN) to detect COVID-19 and community acquired pneumonia (CAP) from CT scans. Bermejo-Peláez et al.^47^ proposed a deep neural network to analyze COVID-19 patterns from CT scans to assess disease severity and predict clinical outcomes. Yao et al.^48^ developed an atrous convolution network to diagnose mild COVID-19 pneumonia from CT scans. Song et al.^49^ developed a Details Relation Extraction neural network to diagnose COVID-19 from CT images. Bratt et al.^50^ developed a deep learning method to predict usual interstitial pneumonia histopathology from CT images. Shiri et al.^51^ developed a deep neural network to assess the severity of COVID-19 based on CT radiomics features. Ortiz^23^ developed a deep neural network which leverages CT scans and clinical metadata to distinguish COVID-19 pneumonia from other viral pneumonia. Existing methods do not consider leveraging external CT data from source domains to mitigate the lack of CT scans that have pneumonia labels or cannot automatically identify and downweight source CT data examples that are noisy and have large domain discrepancy with target domain. Our method bridges these gaps.

### Source data reweighting in transfer learning

Many methods^25–33,52–56^ have been developed to reweight source data examples according to their fitness for training target models, based on bi-level optimization^35,37–40^, reinforcement learning^57–59^, adversarial learning^31^, curriculum learning^27,32^, entropy^60,61^, Bayesian optimization^26^, multi-task learning^25^, etc. In bi-level optimization based methods, an inner optimization problem trains a model on reweighted data and an outer optimization problem learns data weights by minimizing validation loss of the trained model. These methods reweight source data by comparing their similarity with training data in the target domain. As a result, a target model trained using these reweighted source data may overfit to the training data distribution and generalizes poorly on unseen data. Our method reweights source examples by measuring how a target model trained (implicitly) using reweighted source data generalizes to validation examples, and therefore is more robust to overfitting.

### Transfer learning and multi-task learning

The goal of transfer learning (TL)^24,62–66^ is to leverage data in a source domain to help with model training in a target domain. Existing TL methods can be roughly categorized into the following groups: (1) latent space projection^67–70^, (2) distribution alignment^53,71–73^, (3) adversarial domain-invariant representation learning^74–77^, and (4) regularization^78–80^. Multi-task learning (MTL)^81,82^ aims to improve multiple models simultaneously by training them jointly and transferring knowledge across models. Various MTL approaches have been proposed, based on (1) hard parameter sharing^83–87^, where multiple models share the same weight parameters, such as encoder weights; (2) soft parameter sharing^88^, where parameters of different models are constrained to be similar; (3) task similarity learning^89–91^, which identifies similarity between tasks and encourages similar tasks to share more commonalities; (4) loss weighting^87^, which weighs each model’s loss, and so on. Different from previous transfer learning and multi-task learning methods, our method is based on three-level optimization and can automatically identify and downweight source data that is noisy or have large domain discrepancy with target data by minimizing target model’s validation loss.

### Bi-level optimization

Bi-level Optimization (BLO)^92^ has been broadly applied for hyperparameter tuning^93^, neural architecture search^94^, meta learning^95^, data reweighting^37–39^, learning rate adjustment^96^, label denoising^97^, data generation^98^, etc. In these methods, meta parameters (e.g, hyperparameters, neural architectures, data weights, etc.) are optimized by minimining validation losses and model weights are learned by minimizing training losses. Our method goes beyond bi-level optimization and solves a three-level optimization problem for source data reweighting.

## Methods

In this section, we present the method for reweighting source CT data based on three-level optimization. We aim to train a target model $M_t$ to detect pneumonia from CT scans, on a dataset $D_t$ from the target domain which contains CT scans with pneumonia class labels. To mitigate the deficiency of labeled target data, we leverage a CT dataset $D_s$ from a source domain which has pneumonia class labels. A source model $M_s$ is trained on $D_s$. Some examples in $D_s$ are noisy and some examples have large domain discrepancies with $D_t$. We aim to down-weight such low-quality source examples by automatically learning a weight for each source example. For $M_t$, it has an encoder $E_t$ and a head $H_t$. For $M_s$, it has an encoder $E_s$ and a head $H_s$. Knowledge transfer is conducted from $E_s$ to $E_t$. Note that we allow $E_s$ and $E_t$ to have different architectures, different dimensions of encodings, and different weight parameters.

### A three-level optimization framework

**Figure 1:**
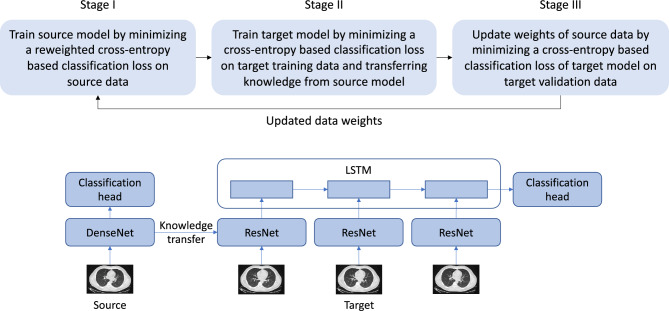
(Top) Overview of the proposed three-level optimization framework. (Bottom) Network architecture of target and source models.

We propose a three-level optimization based framework (Fig. 1(top)) to perform reweighting of source CT data. The framework consists of three learning stages which are performed end-to-end. At the first stage, we train $E_s$ and $H_s$ on $D_s$. For each source example in $D_s=\{d_{s,i}\}_{i=1}^M$, an importance weight $c\in [0,1]$ is to be learned. The training loss *L* of a source example, which is a cross-entropy based classification loss, is multiplied with the importance weight of this example^35^. If *c* is close to 0, it means this example is noisy or has large domain-discrepancy with target data; accordingly, the loss (after multiplied with *c*) is made close to 0, which effectively excludes this example from the training process. We aim to automatically learn these importance weights, which will be detailed later on. This stage amounts to solving the following problem:

1 $$\begin{aligned} \begin{array}{l} E_s^*(C), H_s^*(C)=\text {argmin}_{E_s,H_s}\;\;\sum _{i=1}^{M} c_i L(E_s,H_s, d_{s,i}), \end{array} \end{aligned}$$

where $C=\{c_i\}_{i=1}^{M}$. The importance weights *C* are needed to calculate training losses, but they should not be updated at this stage. Otherwise, the values of *C* will all be zero. Note that $E_s^*(C)$ depends on *C* since it depends on the training loss which is a function of *C*.

At the second stage, we train $E_t$ and $H_t$ on $D_t$ by minimizing a cross-entropy based classification loss *L*. We propose a novel way of transferring knowledge from source model to target model, based on predicting cross-domain relative similarity relationships. Given two target examples, we use the source encoder to label which of them is closer to the source examples. For each target example $x_t$, its distance to source dataset $D_s$ is calculated as $e(x_t,D_s;E_s^*(C))=\text {min}_{d_s\in D_s}c(x_t,d_s;E_s^*(C))$, where $c(x_t,d_s;E_s^*(C))$ is the L2 distance between $x_t$ and a source example $d_s$, both encoded by the source encoder $E_s^*(C)$. For two target examples $x_t$ and $y_t$, if $e(x_t,D_s;E_s^*(C))<e(y_t,D_s;E_s^*(C))$, $x_t$ is labeled as being closer to the source dataset (denoted by $x_t\succ y_t|E_s^*(C))$. The source encoder labels many such pairs. Then these labeled pairs are used to train the target encoder. Given a cross-domain relative similarity (CDRS) relationship between two target examples, the target encoder is trained to predict this relationship. Let $e(x_t,D_s;E_t)$ (defined in a similar way as $e(x_t,D_s;E_s^*(C))$) denotes the distance between $x_t$ and $D_s$, encoded using the target encoder. If $x_t\succ y_t|E_s^*(C)$, then $e(x_t,D_s;E_t)$ is required to be smaller than $e(y_t,D_s;E_t)$. The optimization problem at the second stage is:

$$\begin{aligned} \begin{array}{l} E_t^*(E_s^*(C)), H_t^*= \text {argmin}_{E_t,H_t}\;\; L(E_t,H_t, D_t)\\ \quad s.t. \; \forall x_t\succ y_t|E_s^*(C), e(x_t,D_s;E_t)<e(y_t,D_s;E_t) \end{array} \end{aligned}$$

    CDRS captures the relationships among target examples by generating a global ranking among them, based on their distances to the source domain (such a global ranking can be induced from all pairwise rankings). This global ranking reveals a lot of semantic information of target CT images. For instance, CT images at adjacent positions in the ranking are more similar than those at faraway positions. Grouping similar CT images together using the ranking can facilitate pneumonia classification. In pneumonia classification, there are multiple classes, such as common pneumonia, COVID-19, and no pneumonia. Grouping CT images in the same pneumonia class together based on their positions in the global ranking can better distinguish different classes and improve classification performance.

At the third stage, we use the trained target model consisting of $E_t^*(E_s^*(C))$ and $H_t^*$ to make predictions on a validation dataset $D_t^{({val})}$ in the target domain. We update *C* by minimizing the validation loss *L* which is also a cross-entropy based classification loss.

2 $$\begin{aligned} \text {min}_{C}\;\; L(E_t^*(E_s^*(C)), H_t^*, D_t^{({val})}). \end{aligned}$$

    Putting the three learning stages together, we have the following three-level optimization framework:

3 $$\begin{aligned} \begin{array}{ll} \text {min}_{C}&{} L(E_t^*(E_s^*(C)), H_t^*, D_t^{({val})})\\ \\ s.t. &{} E_t^*(E_s^*(C)), H_t^*= \text {argmin}_{{E_t,H_t}} \;\; L(E_t,H_t, D_t)\\ &{} \quad s.t. \;\; \forall x_t\succ y_t|E_s^*(C), e(x_t,D_s;E_t)<e(y_t,D_s;E_t)\\ \\ &{} \qquad s.t.\;\; E_s^*(C), H_s^*(C)=\text {argmin}_{E_s,H_s}\;\;\sum _{i=1}^{M} c_i L(E_s,H_s, d_{s,i})\\ \end{array} \end{aligned}$$

    The three stages are mutually dependent on each other and are conducted end-to-end. The output of Stage I, which is $E_s^*(C)$, is used as input of Stage II. The outputs of Stage II, which are $E_t^*(E_s^*(C))$ and $H_t^*$, are used as inputs of Stage III. After *C* is updated at Stage III, the objective function in Stage I, which is a function of *C*, will change accordingly, rendering $E_s^*(C)$ to change as well. By solving the three interdependent optimization problems jointly in the multi-level optimization framework, we can perform the three learning stages end-to-end.

The constraint in the above equation is highly discrete, which is not end-to-end differentiable and renders the optimization problem difficult to solve. We address this issue by performing a continuous relaxation of the constraint using pairwise hinge loss:

4 $$\begin{aligned} \begin{array}{l} \lambda \text {max}\bigg (0,-\big (e(x_t,D_s;E_s^*(C))-e(y_t,D_s;E_s^*(C))\big ) \big (e(x_t,D_s;E_t)-e(y_t,D_s;E_t)\big )\bigg ), \end{array} \end{aligned}$$

where $\lambda$ is a tradeoff parameter. Let $p=e(x_t,D_s;E_s^*(C))-e(y_t,D_s;E_s^*(C))$ and $q=e(x_t,D_s;E_t)-e(y_t,D_s;E_t)$. If $pq>0$, the target model predicts the CDRS relationship between $x_t$ and $y_t$ correctly. In this case, the hinge loss is 0 and there is no penalty. Otherwise, the hinge loss is $-pq$, which penalizes the target model to correct its prediction.

### Optimization algorithm

We develop a gradient-based algorithm to solve the three-level optimization problem. Drawing insights from^94^, we approximate $E_s^*(C)$ using one-step gradient descent update of $E_s$:

5 $$\begin{aligned} E_s^*(C)\approx E'_s = E_s-\eta _{es} \nabla _{E_s} \sum _{i=1}^{M} c_i L(E_s,H_s, d_{s,i}), \end{aligned}$$

and update $H_s$ using:

6 $$\begin{aligned} H_s \leftarrow H_s-\eta _{hs} \nabla _{H_s} \sum _{i=1}^{M} c_i L(E_s,H_s, d_{s,i}). \end{aligned}$$

    We plug $E_s^*(C)\approx E'_s$ into the loss function at the second stage and get an approximated objective:

7 $$\begin{aligned} \begin{array}{l} O=L(E_t,H_t, D_t)+ \lambda \sum _{x_t,y_t} \text {max}(0,-(e(x_t,D_s;E'_s)-e(y_t,D_s;E'_s))(e(x_t,D_s;E_t)-e(y_t,D_s;E_t))). \end{array} \end{aligned}$$

    Then we approximate $E_t^*(E_s^*(C))$ and $H_t^*$ using one-step gradient descent update of $E_t$ and $H_t$ w.r.t *O*:

8 $$\begin{aligned} E_t^*(E_s^*(C))\approx E'_t= E_t-\eta _{et} \nabla _{E_t} O, \end{aligned}$$

9 $$\begin{aligned} H_t^*\approx H'_t= H_t-\eta _{ht} \nabla _{H_t} O. \end{aligned}$$

    Finally, we plug these approximations into the validation loss at the third stage and update *C* and $R_s$ by minimizing the approximated loss using gradient descent:

10 $$\begin{aligned} C\leftarrow C-\eta _c \nabla _{C} L(E'_t, H'_t, D_t^{({val})}). \end{aligned}$$

    For $\nabla _{C} L(E'_t, H'_t, D_t^{({val})})$, it can be computed as:

11 $$\begin{aligned} \nabla _{C} L(E'_t, H'_t, D_t^{({val})})=\frac{\partial E'_s}{\partial C} \frac{\partial E'_t}{\partial E'_s} \frac{\partial L(E'_t, H'_t, D_t^{({val})}) }{\partial E'_t}, \end{aligned}$$

where

12 $$\begin{aligned} \frac{\partial E'_t}{\partial E'_s}= -\eta _{et} \nabla ^2_{E'_s,E_t} O, \end{aligned}$$

13 $$\begin{aligned} \frac{\partial E'_s}{\partial C}= -\eta _{es} \nabla ^2_{C,E_s} \sum _{i=1}^{M} c_i L(E_s,H_s, d_{s,i}). \end{aligned}$$

    These steps iterate until convergence. For the calculation of $e(x_t,D_s;E_s^*(C))$, in theory, the complexity is quadratic $O(n^2)$, where *n* is the number of data examples: calculating the distance between each pair of (source, target) examples. In practice, the calculation is conducted on a minibatch of target examples and a minibatch of source examples. The actual complexity is $O(k^2)$ , where *k* is the minibatch size. *k* is at most a few hundred, which is much smaller than *n*. Similar to DARTS^94^, the matrix-vector multiplication in Eq. (11) are approximated using finite differences, which can be calculated efficiently. With this approximation, the complexity is reduced from quadratic to linear (in terms of parameter numbers).

## Dataset

For the target domain dataset, we used the China Consortium of Chest CT Image Investigation (CC-CCII)^99^. It contains 2218 3D CT scans from 557 common pneumonia patients (CP), 682 COVID-19 patients (NCP), and 979 normal controls. The common pneumonia (CP) group includes viral pneumonia (e.g., adenoviral, influenza, and parainfluenza pneumonia), bacterial pneumonia, and mycoplasma pneumonia, which were diagnosed based on standard clinical, radiological, culture/molecular assay results. Novel coronavirus patient (NCP) diagnosis was based on reverse transcriptase PCR. The CTs scans are obtained from Sun Yat-sen Memorial Hospital, Third Affiliated Hospital of Sun Yat-sen University, The first Affiliated Hospital of Anhui Medical University, West China Hospital, Nanjing Renmin Hospital, Yichang Central People’s Hospital, and Renmin Hospital of Wuhan University. The dataset is split into a train, validation, and test set with a ratio of 7:1.5:1.5. For the source domain dataset, we use the one collected by Yang et al.^100^. This dataset contains 2D CT slices extracted from COVID-19 related papers. Each slice is labeled with whether containing COVID-19 findings. It consists of 349 COVID-19 CT images from 216 patients and 463 non-COVID-19 CTs. These CT slices are extracted from 760 preprints about COVID-19 in medRxiv and bioRxiv, posted from Jan 19th to Mar 25th. The minimum, average, and maximum height of these images are 153, 491, and 1853. The minimum, average, and maximum width of these images are 124, 383, and 1485. In this dataset, some images are noisy, annotated with non-clinical artifacts such as bounding boxes and arrows. Some images have large domain differences from target data in terms of resolution, appearance, texture, color, scale, etc.

## Experiments

In this section, we present experimental results.

### Experimental settings

We leverage the proposed three-level optimization framework to reweight the noisy source CT data. The source model performs binary classification on 2D CT slices in the source dataset: given a 2D CT slice, predict whether it contains COVID-19. The source model consists of a 2D image encoder $E_s$ and a classification head $H_s$. The target model performs three-class classification on 3D CT scans in the target dataset, which classifies a 3D CT scan into one of the three classes: NPC, CP, and Normal. The target model consists of an LSTM-CNN encoder^101^ and a classification head $H_t$. The LSTM-CNN encoder is used to encode a 3D CT scan, which contains a sequence of 2D CT slices. For each 2D slice, it is encoded by a CNN encoder $E_t$. Then the sequence of CNN encodings are fed into an LSTM^102^ network $V_t$ to extract a holistic representation of the entire 3D CT scan. The encoding of the 3D CT scan is fed into the classification head to predict the class label. When training $E_t$, we transfer knowledge from $E_s$ to $E_t$.

In the LSTM-CNN target encoder, we set the hidden size to 128 and set the CNN encoder to ResNet-50^103^. For the source encoder, we set it to DenseNet^104^. Dimensions of embeddings generated by the source and target CNN encoders are different. The classification heads $H_t$ and $H_s$ are linear. Figure 1 (bottom) shows the network architecture of source and target models. The tradeoff parameter $\lambda$ was set to 0.1. The initial learning rate was set to 1e-3 with the Adam^105^ optimizer used. The learning rate decayed with cosine scheduling. The momentum for Adam was (0.5, 0.999). The batch size was set to 64. The model was trained for 150 epochs. The dropout^106^ rate was set to 0.3. Weight decay was set to 5e-4. To determine the convergence of the three-level optimization problem, we check the values of the validation loss in the third stage and those of training losses in the first and second stage. For every loss, if the standard deviation of its values in the most recent four epochs is less than 5% of these values’ mean, we consider the algorithm has converged and stop the optimization process.

To tune the hyperparameters, we randomly split the validation set into two equal-sized subsets: denoted by *A* and *B*. For each configuration of hyperparameters, we used the validation set *A* to learn importance weights of source data. Then we measure the performance of the trained model on validation set *B*. Hyperparameter values yielding the best performance on validation set *B* were selected. To ensure a fair comparison, we spent approximately the same time on tuning hyperparameters for each method (including ours and baselines). The tuning time per method is about 16 hours.

Every experiment ran 5 times with different random initializations. Precision, recall, and F1 scores were used as evaluation metrics. For all experiments, we performed significance tests using double-sided t-tests. The p-values of our methods against baselines are all less than 0.001, which shows that our methods are significantly better than baselines. The experiments were conducted on A100 GPU. Our method takes about 26 hours to converge.

### Baselines

We compared our method with the following baselines. The baseline models were trained on the combination of training and validation sets of the target dataset.

- No source data (NoSrc)^101^: we do not leverage source data for model training.
- No reweighting (NoWt)^101^: we use all source data examples for model training without reweighting. NoWt uses CDRS-based knowledge transfer. We first train a source model on all source examples without reweighting, then use this source model to label CDRS relationships. These labeled CDRS relationships are utilized as constraints to train the target model.
- Pretrain^103^: we first pretrain $E_s$ on source data, then use $E_s$ to initialize $E_t$.
- BO^26^: Bayesian optimization based data selection for transfer learning.
- MGTL^31^: a minimax game based model for selective transfer learning.
- Online meta-learning (OML)^35^ for data reweighting: we first unify the formats of 3D CT scans and 2D source CT slices by labeling all slices in a positive/negative CT scan as positive/negative; then on 2D slices, we use an online meta-learning^35^ approach, which is based on bi-level optimization (BLO), to reweight source slices.
- MentorNet^107^: a curriculum learning method for data selection. Similar to OML, format unification is applied.
- Multi-task learning (MTL)^108^: BLO is applied for reweighting source data, where source and target models are trained simultaneously by minimizing the weighted sum of their training losses; source and target encoder weights are encouraged to be similar using L2 regularization to transfer knowledge between source and target encoders and let them help each other to learn.
- Weights sharing (WS)^86^: similar to MTL, except that target and source models share the same encoder.
- We compare our CDRS knowledge transfer approach with: **(1)** L2 regularization on encoder weights (RegW)^109^: encouraging target encoder’s weights to have small L2 distance with source encoder’s weights; **(2)** L2 regularization on embeddings (RegE)^110^: encouraging embeddings generated by target encoder to have small L2 distance with those generated by source encoder; **(3)** pseudo-labeling (PL)^111^: the source model generates pseudo-labels regarding whether CT slices are positive, which are used to train the target model; and **(4)** pairwise similarity (PS)^112^: the source encoder annotates whether two images are similar or dissimilar and the target encoder predicts these similarity labels.
- We also compare with six CT-based pneumonia classification methods: (1) CC-CCII^99^, (2) RapidAI^113^, (3) 3DCNN^11^, (4) Li et al.^114^, (5) Shamsi et al.^115^, and (6) Shaik et al.^116^.

**Table 1:** Precision, recall, and F1 for pneumonia classification from CT scans. Source data is 2D CT slices from COVID-19 related papers. In the $X_Y$ formatted entry, *X* denotes mean and *Y* denotes standard deviation in 5 runs with different random initialization.

	NCP			CP			Normal		
	Precision (%)	Recall (%)	F1 (%)	Precision (%)	Recall (%)	F1 (%)	Precision (%)	Recall (%)	F1 (%)
CC-CCII^99^	84.5$_{.5}$	87.2$_{.7}$	85.8$_{.4}$	88.9$_{.8}$	87.5$_{.6}$	88.2$_{.2}$	85.9$_{.6}$	86.3$_{.7}$	86.1$_{.7}$
RapidAI^113^	83.1$_{.2}$	86.8$_{.5}$	84.9$_{.1}$	86.3$_{.5}$	86.0$_{.4}$	86.1$_{.3}$	83.8$_{.8}$	85.5$_{.6}$	84.6$_{.8}$
3D-CNN^11^	86.6$_{.7}$	87.2$_{.5}$	86.9$_{.5}$	85.4$_{.7}$	87.7$_{.9}$	86.5$_{.7}$	86.8$_{.5}$	85.9$_{.9}$	86.3$_{.2}$
Li et al.^114^	84.2$_{.3}$	88.1$_{.6}$	86.1$_{.4}$	89.4$_{.4}$	88.5$_{.3}$	88.9$_{.3}$	86.6$_{.7}$	87.2$_{.4}$	86.9$_{.5}$
Shamsi et al.^115^	85.4$_{.5}$	87.9$_{.4}$	86.6$_{.5}$	90.1$_{.6}$	89.1$_{.6}$	89.6$_{.5}$	88.5$_{.8}$	88.9$_{.5}$	88.7$_{.7}$
Shaik et al.^116^	84.9$_{.3}$	88.7$_{.9}$	86.8$_{.5}$	88.2$_{.8}$	88.3$_{.2}$	88.2$_{.6}$	87.2$_{.3}$	87.4$_{.5}$	87.3$_{.5}$
NoSrc^101^	85.9$_{.9}$	87.5$_{.6}$	86.7$_{.6}$	88.1$_{.9}$	89.2$_{.6}$	88.6$_{.9}$	86.2$_{.9}$	87.1$_{.9}$	86.6$_{.5}$
NoWt^101^	86.4$_{.7}$	87.1$_{.4}$	86.7$_{.4}$	88.6$_{.4}$	89.8$_{.5}$	89.2$_{.7}$	88.1$_{.3}$	88.5$_{.8}$	88.3$_{.2}$
Pretrain^103^	88.6$_{.4}$	90.1$_{.8}$	89.3$_{.3}$	89.1$_{.5}$	90.9$_{.4}$	90.0$_{.2}$	88.6$_{.7}$	87.2$_{.5}$	87.9$_{.7}$
BO^26^	87.3$_{.6}$	88.5$_{.3}$	87.9$_{.8}$	89.2$_{.9}$	90.5$_{.8}$	89.8$_{.6}$	89.2$_{.2}$	88.5$_{.6}$	88.8$_{.4}$
MGTL^31^	87.5$_{.5}$	90.7$_{.6}$	89.1$_{.6}$	90.2$_{.7}$	91.7$_{.3}$	90.9$_{.5}$	88.5$_{.6}$	89.3$_{.4}$	88.9$_{.9}$
OML^35^	87.1$_{.8}$	88.4$_{.8}$	87.7$_{.8}$	89.5$_{.4}$	89.7$_{.9}$	89.6$_{.5}$	88.1$_{.4}$	88.3$_{.8}$	88.2$_{.1}$
MentorNet^107^	87.6$_{.6}$	88.2$_{.2}$	87.9$_{.4}$	89.8$_{.6}$	90.3$_{.7}$	90.0$_{.7}$	88.4$_{.4}$	89.1$_{.5}$	88.7$_{.5}$
MTL^108^	89.1$_{.4}$	90.7$_{.7}$	89.9$_{.7}$	90.9$_{.5}$	91.2$_{.4}$	91.0$_{.5}$	88.2$_{.6}$	88.7$_{.7}$	88.4$_{.8}$
WS^86^	89.2$_{.3}$	89.7$_{.7}$	89.4$_{.4}$	90.3$_{.6}$	91.1$_{.5}$	90.7$_{.8}$	87.3$_{.7}$	88.1$_{.8}$	87.7$_{.2}$
No-LSTM	90.0$_{.4}$	91.4$_{.6}$	90.7$_{.5}$	91.3$_{.3}$	92.0$_{.4}$	90.2$_{.4}$	89.2$_{.5}$	90.3$_{.3}$	90.1$_{.5}$
Same-ResNet	90.5$_{.2}$	89.9$_{.9}$	91.6$_{.5}$	90.8$_{.9}$	91.4$_{.5}$	91.1$_{.8}$	89.4$_{.7}$	90.1$_{.4}$	90.9$_{.5}$
Same-DenseNet	90.3$_{.5}$	90.0$_{.6}$	89.7$_{.6}$	90.5$_{.2}$	91.3$_{.8}$	89.7$_{.6}$	89.4$_{.4}$	89.0$_{.7}$	89.2$_{.5}$
Ours+RegW^109^	88.9$_{.2}$	91.0$_{.3}$	89.9$_{.2}$	91.1$_{.4}$	89.8$_{.3}$	90.4$_{.6}$	87.8$_{.4}$	89.2$_{.6}$	88.5$_{.9}$
Ours+RegE^110^	88.6$_{.5}$	89.8$_{.5}$	89.2$_{.1}$	91.3$_{.2}$	90.1$_{.6}$	90.7$_{.4}$	87.3$_{.3}$	90.2$_{.5}$	88.7$_{.5}$
Ours+PL^111^	89.2$_{.3}$	90.4$_{.8}$	89.8$_{.9}$	90.7$_{.5}$	91.4$_{.4}$	91.0$_{.3}$	88.7$_{.5}$	87.9$_{.6}$	88.3$_{.6}$
Ours+PS^112^	89.5$_{.4}$	88.6$_{.6}$	89.0$_{.1}$	90.8$_{.3}$	89.9$_{.5}$	90.3$_{.3}$	88.9$_{.8}$	88.1$_{.4}$	88.5$_{.2}$
Ours+CDRS	91.4$_{.2}$	92.2$_{.2}$	91.8$_{.1}$	92.1$_{.2}$	92.8$_{.3}$	92.4$_{.8}$	90.3$_{.3}$	91.5$_{.4}$	90.9$_{.7}$

**Figure 2:**
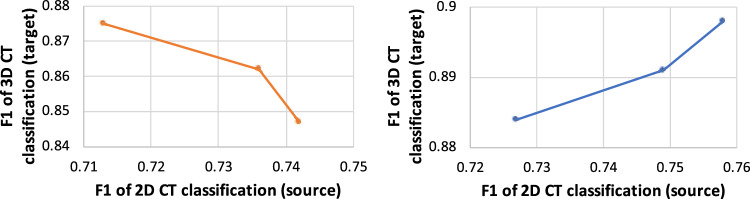
(Left) Evidence of model competition in MTL. (Right) How the performance of the target model varies with the performance of the source model in our framework.

**Figure 3:**
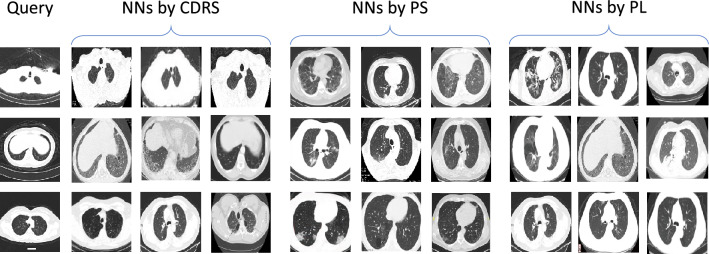
4-nearest source neighbors retrieved by different methods for some randomly sampled target CT slices. Compared with PS and PL, nearest neighbors retrieved by our CDRS method are more semantically similar to query slices. The similarity is evaluated by physicians and is determined based on whether clinical findings in two images are clinically close.

### Results and analysis

Table 1 shows the results. Our method achieves an F1 score of 91.8% in detecting COVID-19 pneumonia (NCP) and an F1 score of 92.4% in detecting other types of pneumonia (CP). Our method performs better than NoSrc. In NoSrc, no source data is leveraged for learning representations, which is a waste. Many source 2D CT slices contain abnormalities related to COVID-19. An encoder trained using these CT slices can learn representations capturing such abnormalities, which is helpful for classifying COVID-19. Our method performs better than NoWt. In NoWt, all source examples are used without reweighting. Many source examples are noisy or incorrectly labeled. Trained using such low-quality data, the source encoder $E_s$ may learn poor representations. Transferring low-quality representations from $E_s$ to $E_t$, the effectiveness of $E_t$ may be degraded as well, which yields inferior performance in classifying 3D CT scans. In contrast, our method performs reweighting of each source data example by checking whether it can help to reduce the validation loss of the target model. If a source example hurts classification performance on 3D CT scans, our method automatically assigns a small weight to it. Our method outperforms state-of-the-art methods developed for pneumonia classification, including CC-CCII^99^, RapidAI^113^, 3DCNN^11^, Li et al.^114^, Shamsi et al.^115^, and Shaik et al.^116^. The reason is that these methods do not leverage auxiliary source data or lack capability of reweighting source data.

**Table 2:** Precision, recall, and F1 for pneumonia classification from CT scans. Source data is 3D CT scans. In the $X_Y$ formatted entry, *X* denotes mean and *Y* denotes standard deviation in 5 runs with different random initialization.

	NCP			CP			Normal		
	Precision (%)	Recall (%)	F1 (%)	Precision (%)	Recall (%)	F1 (%)	Precision (%)	Recall (%)	F1 (%)
CC-CCII^99^	84.5$_{.5}$	87.2$_{.7}$	85.8$_{.4}$	88.9$_{.8}$	87.5$_{.6}$	88.2$_{.2}$	85.9$_{.6}$	86.3$_{.7}$	86.1$_{.7}$
RapidAI^113^	83.1$_{.2}$	86.8$_{.5}$	84.9$_{.1}$	86.3$_{.5}$	86.0$_{.4}$	86.1$_{.3}$	83.8$_{.8}$	85.5$_{.6}$	84.6$_{.8}$
3D-CNN^11^	86.6$_{.7}$	87.2$_{.5}$	86.9$_{.5}$	85.4$_{.7}$	87.7$_{.9}$	86.5$_{.7}$	86.8$_{.5}$	85.9$_{.9}$	86.3$_{.2}$
Li et al.^114^	86.2$_{.5}$	82.3$_{.4}$	84.2$_{.5}$	90.9$_{.7}$	90.3$_{.4}$	90.6$_{.5}$	89.4$_{.4}$	87.9$_{.7}$	88.6$_{.6}$
Shamsi et al.^115^	86.4$_{.3}$	87.4$_{.5}$	86.9$_{.6}$	91.7$_{.3}$	89.9$_{.5}$	90.8$_{.4}$	90.4$_{.6}$	89.2$_{.3}$	89.8$_{.5}$
Shaik et al.^116^	87.0$_{.6}$	89.6$_{.3}$	88.3$_{.4}$	89.7$_{.5}$	89.1$_{.4}$	89.4$_{.4}$	87.8$_{.2}$	87.7$_{.4}$	87.8$_{.3}$
NoSrc^101^	85.9$_{.9}$	87.5$_{.6}$	86.7$_{.6}$	88.1$_{.9}$	89.2$_{.6}$	88.6$_{.9}$	86.2$_{.9}$	87.1$_{.9}$	86.6$_{.5}$
NoWt^101^	89.1$_{.4}$	86.6$_{.5}$	87.8$_{.5}$	88.2$_{.6}$	90.7$_{.8}$	89.4$_{.6}$	90.4$_{.5}$	91.2$_{.3}$	90.8$_{.3}$
Pretrain^103^	89.9$_{.7}$	92.5$_{.2}$	91.2$_{.6}$	89.6$_{.3}$	91.5$_{.8}$	90.5$_{.6}$	87.9$_{.4}$	88.5$_{.7}$	88.2$_{.6}$
BO^26^	89.6$_{.3}$	89.8$_{.6}$	89.7$_{.4}$	91.5$_{.5}$	92.1$_{.4}$	91.8$_{.5}$	89.9$_{.4}$	87.6$_{.3}$	88.7$_{.3}$
MGTL^31^	90.3$_{.8}$	93.4$_{.3}$	91.8$_{.5}$	91.2$_{.4}$	91.9$_{.5}$	91.5$_{.4}$	90.8$_{.9}$	90.3$_{.6}$	90.5$_{.7}$
OML^35^	88.3$_{.4}$	88.9$_{.6}$	88.6$_{.5}$	90.5$_{.7}$	91.3$_{.5}$	90.9$_{.6}$	88.3$_{.7}$	89.5$_{.2}$	88.9$_{.4}$
MentorNet^107^	87.1$_{.4}$	88.7$_{.3}$	87.9$_{.3}$	90.6$_{.7}$	91.1$_{.4}$	90.8$_{.5}$	89.3$_{.1}$	90.5$_{.8}$	89.9$_{.6}$
MTL^108^	91.4$_{.6}$	91.2$_{.4}$	91.3$_{.5}$	90.3$_{.9}$	91.9$_{.1}$	91.1$_{.7}$	90.6$_{.8}$	88.3$_{.3}$	89.4$_{.5}$
WS^86^	90.4$_{.8}$	90.3$_{.4}$	90.4$_{.5}$	91.7$_{.3}$	91.8$_{.7}$	91.8$_{.5}$	89.9$_{.5}$	89.4$_{.3}$	89.6$_{.4}$
Ours	93.7$_{.4}$	94.6$_{.3}$	94.1$_{.3}$	93.1$_{.4}$	93.6$_{.6}$	93.3$_{.5}$	91.6$_{.5}$	92.9$_{.4}$	92.2$_{.3}$

Our method outperforms MTL. In MTL, target model and source model are trained simultaneously by minimizing the weighted sum of their loss functions. In our experiments, we found that these two models have a competing relationship during training: improving performance of one model incurs performance degradation of the other model. Figure 2(left) shows how test performances of source and target models vary with the weight $\beta$ of source model’s training loss (the weight of target model’s training loss is set to 1). The source model is evaluated on a clean 2D CT test set. As $\beta$ increases (more attention is paid to minimizing the source model’s loss), F1 of source model increases while F1 of target model decreases. This demonstrates that MTL incurs a competition between the two models. Our method addresses this problem by training these two models in two different optimization problems in an end-to-end framework. We first train the source model, then leverage transfer learning to train the target model. In this way, the source model helps the target model to learn, instead of competing with it. We provide some empirical evidence by doing the following experiments. First, we train three source encoders that have different accuracy. Three clean 2D CT validation sets with increasing example numbers and one clean 2D test set are collected. Using each validation set, we learn training data weights based on the method proposed by Ren et al.^35^ and train a model accordingly. Using these learned source models as regularization, we train three target models. Figure 2(right) shows that the performance of target models increases when the test performance of source models (used for regularization) increases. This shows that a better source model yields a better target model in our method.

**Figure 4:**
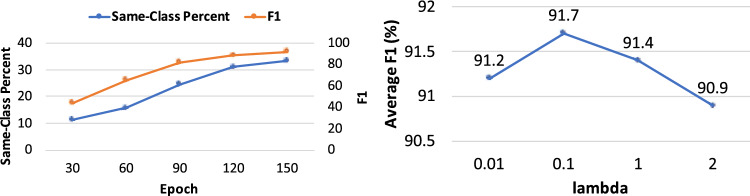
(Left) How same-class percentage and F1 change with epochs in our method. (Right) How the performance of Ours+CDRS changes with $\lambda$.

**Figure 5:**
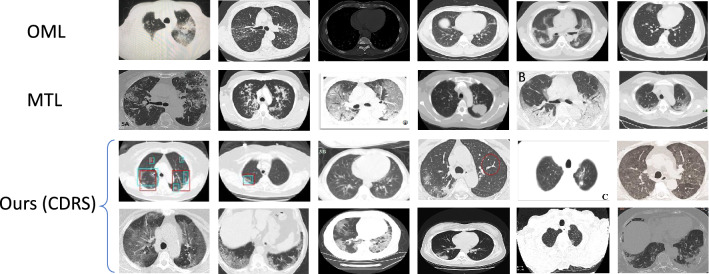
Randomly-sampled source images whose importance weights learned by different methods are close to 0. Our method can successfully identify images containing artifact noises such as bounding boxes or having large domain discrepancies with target data in terms of appearance, texture, color, scale, etc. In contrast, OML and MTL incorrectly assign close-to-zero weights to some images that are clean and have large domain similarity to target data.

Our method works better than RegE and PL. These two baselines require embeddings learned by source and target models to be similar in an absolute sense (L2 difference or KL divergence), which is more restrictive than requiring embeddings to preserve ranking as our method does. Besides, these two baselines transfer knowledge on individual data instances without considering the relationship between instances. In contrast, our method compares which target slice is more close to the source dataset during knowledge transfer. Our method works better than PS. PS is limited to capturing second-order relationships among instances while each piece of knowledge in our CDRS method involves two target instances and all source instances, which therefore can capture higher-order relationships. Figure 3 shows 4-nearest source neighbors retrieved by different methods for some randomly sampled target CT slices. As can be seen, compared with PS and PL, nearest neighbors retrieved by CDRS are more semantically similar to query slices. The similarity is evaluated by physicians and is determined based on whether clinical findings in two images are clinically close. A better ability of comparing source and target slices can help to better identify low-quality source data: if a source slice is very different from target slices, it is likely to be noisy or out of target domain. Our method works better than RegW. RegW requires target and source encoders to have the same architecture so that the distance between their weights can be calculated. This requirement prohibits learning representations tailored to specific datasets. 2D CT slices in CC-CCII have different properties than those in COVID-Papers. CC-CCII CTs are obtained from medical imaging databases in hospitals while COVID-Papers CTs are extracted from PDF-format papers. Using the same encoder architecture to represent them fails to account for such differences. In contrast, our method allows different encoders to have different architectures. Our method performs better than WS. In WS, target and source models share the same 2D CT encoder, which prohibits the learning of dataset-specific representations. We performed an ablation study to further investigate whether it is beneficial to let the 2D CT slice encoders in source and target models have different architectures. Specifically, we set the two encoders in Ours+CDRS to be 1) both ResNet-50, and 2) both DenseNet. Table 1 shows the results of these two ablation settings denoted as Same-ResNet and Same-DenseNet. As can be seen, these two ablation settings perform worse than Ours+CDRS which uses different architectures for the two encoders. This further demonstrates that it is beneficial to use different architectures for source and target encoders to learn dataset-specific representations.

Our method works better than BO and MGTL. These two methods reweight source data without considering the performance of the target model on a held-out validation set, which leads to worse generalization performance on test data. Our method outperforms OML and MentorNet. In OML and MentorNet, 3D CT scans are reduced to 2D slices for the sake of making target data have compatible format as source data. As a result, temporal information in 3D scans is lost, which leads to worse classification performance. In OML and MentorNet, format compatibility is required on both input data and output labels where the input data needs to be 2D CT slices and the output label is COVID/Non-COVID. To meet such a requirement, when reducing 3D CT scans into 2D slices, each slice needs to be given a COVID/Non-COVID label using heuristics: every 2D slice in a COVID-positive 3D CT scan is labeled as COVID. This heuristic is noisy: it could be possible that some 2D slices in a COVID-positive 3D scan do not contain COVID-related abnormalities. In our method, when calculating $e(x_t,D_s;E^*_s(C))$, format compatibility is only required on input data, not on output labels. Therefore, our method does not suffer from the noisy labeling problem of OML and MentorNet. Our method performs better than Pretrain. This is because Pretrain learns source model and target model separately while our method trains these two models jointly end-to-end.

We also performed experiments where a 3D CT scan dataset^117^ is used as source data. It contains 753 CT scans of COVID-19 patients. The architecture of the source model in our method is set to be the same as that of the target model described in the Experimental Settings section. Hyperparameters are the same as those described in the Experimental Settings section. For the OML baseline, no format unification between source and target data is needed. Table 2 shows the results. Our method outperforms all baselines. The analysis of reasons is similar to that for results in Table 1.

We also investigated whether the global ranking (of target CT images) generated in our proposed CDRS approach can group images with the same class together. We calculated the percentage of adjacent images (in the ranking) that have the same class label for model checkpoints of our method at different epochs. Figure 4(left) shows how this same-class percentage varies with training epochs, together with each checkpoint’s average F1 of the three classes on test data. As can be seen, as our method runs for more epochs, the same-class percentage increases. This demonstrates that our method can encourage images from the same class to be grouped together in the global ranking. In addition, test F1 increases as the same-class percentage increases. This demonstrates that a better grouping of same-class images in the global ranking facilitates pneumonia classification.

We performed an ablation study on the LSTM component in the target model, by replacing it with an averaging operation: instead of feeding the representations of CT slices into the LSTM network, we average these representations and feed the averaged representation into the classification head. Table 1 shows the results of this ablation setting (denoted as No-LSTM). The performance of No-LSTM is worse than Ours+CDRS which uses LSTM. This is because No-LSTM cannot capture the sequential relationship between CT slices while LSTM can.

Figure 4(right) shows how the performance (average F1 of NCP, CP, and Normal) of Ours+CDRS changes with $\lambda$ in Eq. (4). As can be seen, a $\lambda$ value in the middle ground yields the best performance. If $\lambda$ is too small (e.g., 0.01), there is not sufficient knowledge transfer from source to target. If $\lambda$ is too large (e.g., 2), the target encoder is excessively influenced by the source encoder and therefore is less capable of learning representations that are tailored to the target dataset.

Figure 5 shows some source examples whose importance weights learned by our method are close to 0 (indicating these examples are noisy or have large domain discrepancy with target data). The range of importance weights after optimization is between [0,1]. As can be seen, some of these examples are indeed noisy. For instance, some contain artifacts such as bounding boxes. In some examples, lung regions are distorted. Though images on the last row do not contain obvious artifact noise, their appearance, texture, color, scale, and positions of lungs are different from those in the target dataset. This shows that our method is not only able to identify obviously noisy source examples, but also those having domain discrepancy with target data. MTL and OML incorrectly assign close-to-zero weights to some images that are clean and have large domain similarity to target data. Another finding is that our method tends to give COVID-19 CTs more weight to make the two classes (COVID and Non-COVID) more balanced. Without reweighting, the ratio between these two classes is about 0.75. After reweighting, the ratio is 0.84, getting closer to 1. As a result, performance of different classes in the 3D CT scan test set is more balanced. Without reweighting (NoWt), the ratio of F1 scores achieved on NCP, CP, and Normal is 1:1.03:1.02. After reweighting (ours), the ratio becomes 1:1.01:0.99.

**Figure 6:**
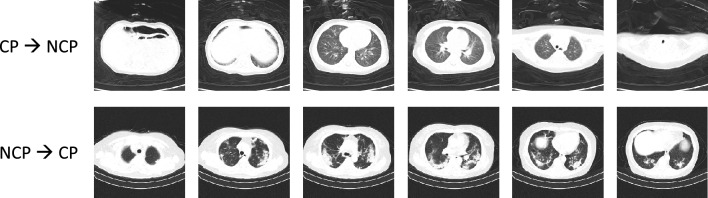
Two failure cases of our method. Each row contains some slices of a target CT scan. The first CT scan is from the CP class, but is incorrectly predicted as being from the NCP class. The second CT scan is from the NCP class, but is incorrectly predicted as being from the CP class. The cause of failure is that the two types of pneumonia have similar findings in CTs, which makes it challenging to distinguish them.

Figure 6 shows two failure cases of our method. Each row contains some slices of a target CT scan. The first CT scan is from the CP class, but is incorrectly predicted as being from the NCP class. The second CT scan is from the NCP class, but is incorrectly predicted as being from the CP class. The cause of failure is that the two types of pneumonia have similar findings in CTs, which makes it challenging to distinguish them.

## Conclusions

To accurately detect pneumonia from CT scans and differentiate COVID-19 and other types of pneumonia, we propose a three-level optimization based method which leverages CT images from a source domain to improve the training of a target model. Our method automatically identifies and down-weights low-quality source CT data examples that are noisy or have large discrepancies with target domain, by checking whether a source example is helpful in reducing validation loss of the target model. Our framework involves three learning stages. At the first stage, we train a source model on weighted source data. At the second stage, by transferring knowledge from source model to target model, we train a target model. We propose a novel knowledge transfer approach based on cross-domain relative similarity. At the third stage, we learn the importance weights of source examples by minimizing the validation loss of the target model. The three stages are performed end-to-end. Our method achieves an F1 score of 91.8% in detecting COVID-19 and an F1 score of 92.4% in detecting other types of pneumonia and outperforms state-of-the-art baselines significantly.

## Data Availability

The China Consortium of Chest CT Image Investigation (CC-CCII) is used as target dataset, which is available at http://ncov-ai.big.ac.cn/download?lang=en The source dataset is available at https://github.com/UCSD-AI4H/COVID-CT and https://wiki.cancerimagingarchive.net/display/Public/CT+Images+in+COVID-19#702271073df18fec0c954449874468eec045f67d
